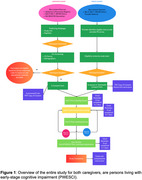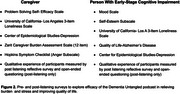# Podcaring: Feasibility Study Design in Implementing the Dementia Untangled Podcast as an Intervention to Relieve Stress and Burden and Improve Quality of Life

**DOI:** 10.1002/alz70858_107510

**Published:** 2025-12-26

**Authors:** Heather Mulder, David W. Coon, Nellie M. High, Robert J. Bauer, Jeremy J. Pruzin

**Affiliations:** ^1^ Banner Alzheimer's Institute, Phoenix, AZ, USA; ^2^ Arizona State University, Phoenix, AZ, USA

## Abstract

**Background:**

Caregivers for persons with neurodegenerative disease experience high rates of stress and burden resulting in worse outcomes for patients. Next, people living with cognitive impairment tend to experience public and self‐stigmatization. Educational interventions that are sensitive to the unique constraints of people and caregivers for persons with neurodegenerative disease, that are broadly accessible, and are delivered in a convenient and modern medium may help lower caregiver burden and improve quality of life for both patients and caregivers. Dementia Untangled is a podcast offering expert guidance and support for people living with cognitive impairment and their care partners, focusing on innovative ideas, practical strategies, and proven methods in an approachable, conversational format. A formative evaluation and preliminary research are needed to determine if this unique delivery method might be feasible as a structured intervention to maximize its impact.

**Method:**

We developed a feasibility study investigating the Dementia Untangled podcast as a tool to potentially relieve stress and burden and improve quality of life for both caregivers and persons with early‐stage cognitive impairment (PWESCI), either mild cognitive impairment or mild dementia. A web‐based clinical trial will examine the feasibility of conducting recruitment, task fulfillment, and evaluate the qualitative experience of caregivers and PWESCI. Both groups will be asked to complete pre‐listening surveys, listen to 8 (caregivers) or 4 (PWESCI) prespecified episodes of Dementia Untangled over two months, and then complete post‐listening surveys. We will explore efficacy of the podcast as measured by the change on pre compared to post‐listening surveys. A diagram of the study design is shown in Figure 1. The specific scales used for each group are shown in Figure 2.

**Result:**

The protocol is now institutional review board approved and the web‐based study has been bult using REDCap. Recruitment will begin in February, 2025. Enrollment goals are 100 caregivers and 30 PWESCI.

**Conclusion:**

Data from this study could support the design and application for expanded and more rigorous clinical testing of the podcast in a clinical trial, with the ultimate goal of adding the Dementia Untangled podcast as a non‐pharmacological tool for more comprehensive dementia care for both caregivers and PWESCI.